# Association between the atherogenic index of plasma and non-alcoholic fatty liver disease in Korean pregnant women: secondary analysis of a prospective cohort study

**DOI:** 10.3389/fnut.2025.1511952

**Published:** 2025-01-31

**Authors:** Rong Shuai, Yuxing He, Dongqian Yang, Yingying Zhang, Li Zhang

**Affiliations:** ^1^Department of Laboratory Medicine, Changde Hospital, Xiangya School of Medicine, Central South University (The First People’s Hospital of Changde City), Changde, China; ^2^Department of Laboratory Medicine, Affiliated Wuxi Fifth Hospital of Jiangnan University, Wuxi, China

**Keywords:** secondary analysis, atherogenic index of plasma, non-alcoholic fatty liver disease, logistic regression model, sensitivity analysis

## Abstract

**Background:**

Recent studies have shown an association between atherogenic index of plasma (AIP) and nonalcoholic fatty liver disease (NAFLD), but the association in a population of pregnant women remains unclear.

**Objectives:**

Our study aimed to examine the association between AIP and NAFLD in pregnant Korean women.

**Methods:**

Our study used publicly available data from Korea, which recruited singleton pregnant women between November 2014 and September 2016 who were at 10–14 weeks of gestation. The presence of NAFLD was diagnosed by liver ultrasound. AIP was calculated as log10 (TG/HDL). Participants were grouped according to AIP tertile: T1 (< 0.16, *n* = 195), T2 (0.16–0.32, *n* = 195), and T3 (>0.32, *n* = 196). Logistic regression models were used to estimate the relationship between AIP and NAFLD. Subgroup and sensitivity analyses were conducted to explore the stability of this relationship. Restricted cubic spline (RCS) curve fitting was employed to investigate potential non-linear associations.

**Results:**

After excluding data on missing variables, 586 singleton pregnant women were finally included. The subjects included in the study had an average AIP of 0.22 (0.11, 0.37), and NAFLD occurred in 110 (18.8%) pregnant women. We observed a positive linear association between AIP and NAFLD (OR = 1.33, 95% CI: 1.19–1.48), which persisted after adjusting for potential confounders (OR = 1.2, 95% CI: 1.06–1.37). When AIP was used as a categorical variable, after adjusting for covariates, the NAFLD risk was significantly higher in the highest tertile of AIP than in the lowest group (OR = 2.02, 95% CI: 1.11–3.68). Their correlations were stable across subgroups and sensitivity analyses.

**Conclusion:**

In this secondary analysis of a prospective cohort study of pregnant Korean women, AIP was found to be positively associated with NAFLD. These outcomes might be used to screen for NAFLD in pregnant women.

## 1 Introduction

Nonalcoholic fatty liver disease (NAFLD) is a prevalent chronic liver condition globally, affecting approximately 25.24% of cases (1). It is defined as the abnormal accumulation of fat within the liver in the absence of viral hepatitis, hepatobiliary disease, or excessive alcohol consumption, and is one of the most common liver diseases in adults (2). It has the potential to progress to cirrhosis and liver cancer (3, 4). NAFLD is frequently associated with obesity, diabetes, hypertension, and metabolic syndrome (5–7). Based on weighted discharge data from the National Inpatient Sample in the United States, the incidence of NAFLD during pregnancy nearly doubled, increasing from 10.5 cases per 100,000 pregnancies in 2007 to 28.9 cases per 100,000 pregnancies in 2015 (8), while NAFLD is also the leading cause of cirrhosis in pregnancy (9, 10). Moreover, recent studies have identified a link between NAFLD in pregnant women and an elevated risk of gestational diabetes and fetal overgrowth, which can have adverse consequences for both the mother and the fetus, as well as the newborn (11, 12). Dyslipidemia is commonly observed in women during pregnancy (13), particularly in the middle and late stages, when blood lipids experience a substantial increase (14–16). In most cases, this is a necessary physiological adjustment to meet the energy needs of the fetus, nevertheless, if the threshold is surpassed, NAFLD may occur. As a result, early detection of NAFLD in pregnant women is crucial for decreasing negative pregnancy outcomes and arresting metabolic disorders.

The Atherogenic Index of Plasma (AIP), derived from the logarithm of the triglyceride (TG) to high-density lipoprotein (HDL) cholesterol ratio, is considered a reliable indicator of dyslipidemia, obesity, and cardiovascular disease (17). Dyslipidemia is closely linked to insulin resistance, with AIP serving as a critical connection. Moreover, the inactivation of sirtuin 1 (SIRT1) has been associated with the progression of croNAFLD; thus, AIP may promote the development of NAFLD by modulating SIRT1 activity (18, 19). AIP also shows significant correlations with various biomarkers, demonstrating negative associations with Vaspin and 25-(OH)D3 (20), and positive correlations with liver enzymes (such as alanine aminotransferase and aspartate aminotransferase) and indicators of insulin resistance (such as homeostasis model assessment-insulin resistance) (21, 22). These associations suggest that AIP may influence the pathological processes of NAFLD through these biomarkers. As a comprehensive index of blood lipids, AIP has been shown to predict the size of lipoprotein particles effectively. Additionally, several studies have demonstrated that AIP outperforms conventional lipid parameters, such as TG, total cholesterol (TC), and low-density lipoprotein (LDL), in predicting NAFLD (23). The AIP is closely related not only to NAFLD but also to metabolic syndrome, cardiovascular disease, and other metabolic abnormalities, suggesting it may serve as a more comprehensive indicator of lipid metabolism. Furthermore, AIP has been observed to be particularly pronounced in women and lean individuals (24), indicating its enhanced diagnostic value in specific populations. The fat metabolism of pregnant women is complex, and there are few studies that have investigated the relationship between AIP and NAFLD in this special population. Therefore, this study aimed to explore the relationship between AIP, both as a continuous and categorical variable and NAFLD in pregnant women, in order to ascertain whether AIP can be utilized as a screening tool to assess the risk of NAFLD within this population.

## 2 Methods

### 2.1 Study design and data source

The original study was a prospective cohort study conducted by Korean researchers, the “Fatty Liver in Pregnancy” registry (NCT02276144), and we conducted secondary analyses using their publicly available data. They recruited singleton pregnant women who received antenatal care before 14 weeks of gestation at Seoul Women’s Hospital in Incheon and Seoul National University Boramae Medical Center in Seoul Metropolitan Government between November 2014 and September 2016 to determine the risk of NAFLD on pregnancy outcomes. The original study ethics were approved by the Seoul National University Boramae Medical Centre Institutional Review Board and the Public Institutions Review Board of the Ministry of Health and Welfare of Korea (25), and no further ethical authorisation was required as we were a secondary analysis. In addition, the original study followed the principles of the Declaration of Helsinki, and each pregnant participant signed an informed consent form. The original data were published in the article “Nonalcoholic fatty liver disease is a risk factor for large-for-gestational-age birthweight” in PLoS ONE (25). These data are under the Creative Commons Attribution License, which permits unrestricted use, distribution, and reproduction as long as the author and source are properly credited. The contributors of the data are gratefully acknowledged.

### 2.2 Study population

**Figure 1:**
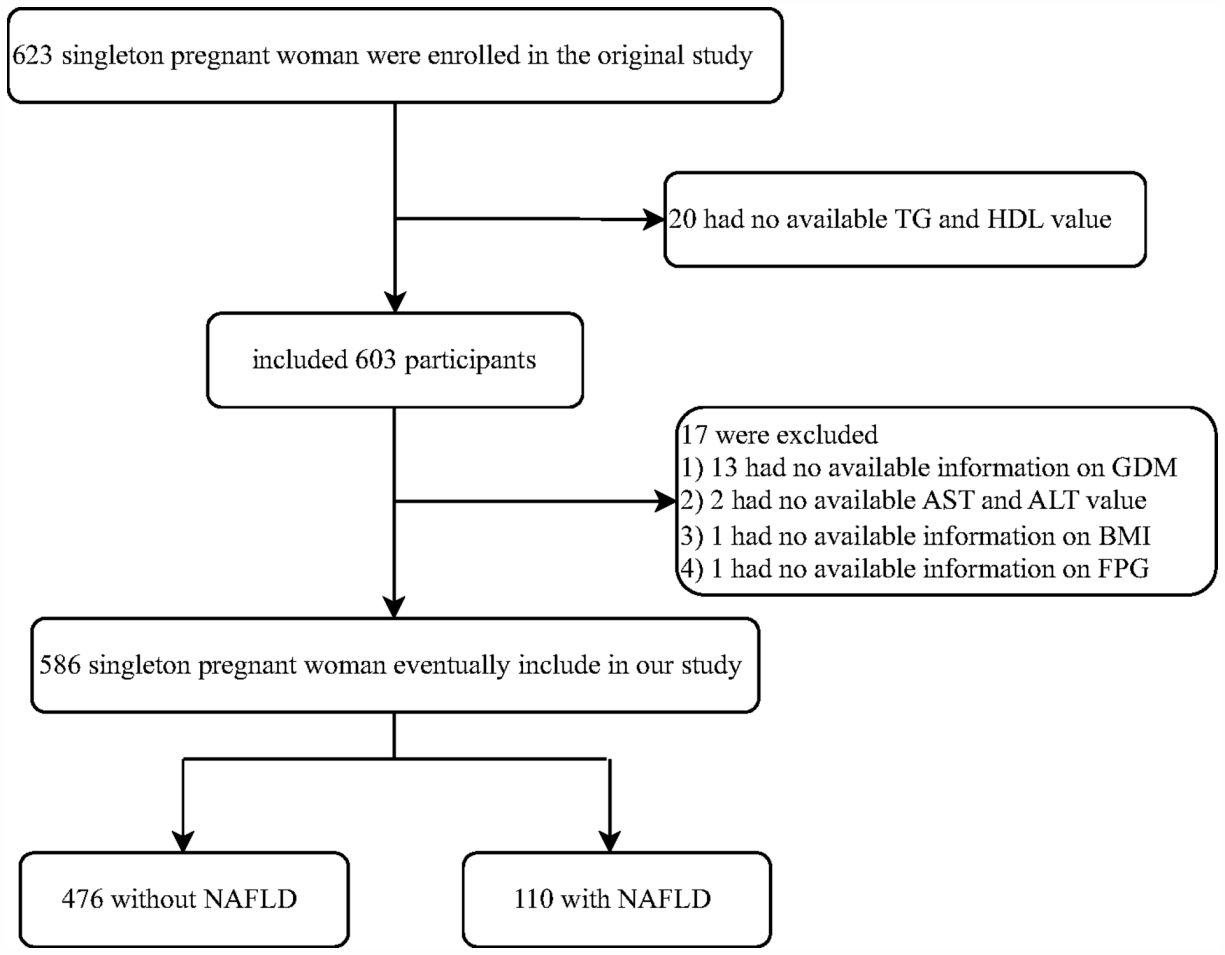
Flowchart of study participants.

The original experiment included 623 singleton pregnant women without chronic liver disease (such as hepatitis B or C, autoimmune hepatitis, primary biliary cholangitis), preconception diabetes, or high alcohol intake. 37 individuals were further excluded from our study, and 586 singleton pregnant women were finally included in our analyses due to missing exposure variables (TG and HDL, *n* = 20) and covariates (*n* = 17) (Figure 1).

### 2.3 Data collection

During the trial, individuals provided venous blood samples following a minimum 8-h fasting interval between 10 and 14 weeks gestation. The samples were tested for biomarkers such as aspartate aminotransferase (AST), alanine aminotransferase (ALT), gamma-glutamyl transferase (GGT), TC, TG, HDL cholesterol, LDL cholesterol, fasting glucose (FPG), and insulin. General demographic and clinical information about the pregnant mother, including her age, quantity of prior births, pre-pregnancy body mass index (BMI), alcohol intake, history of diabetes, and history of chronic liver disease (such as Hepatitis B or Hepatitis C, Autoimmune Hepatitis and Primary Biliary Cholangitis), was collected by a health professional. Maternal liver ultrasounds were conducted by an experienced sonographer between 10 and 14 weeks of gestation. Additionally, a screening test for gestational diabetes mellitus (GDM) was performed on each participant between 24 and 28 weeks of gestation (26, 27). Thanks to the contributors who supported the data collection. Age, parity, BMI, AST, ALT, GGT, TC, TG, HDL cholesterol, LDL cholesterol, FPG, insulin, GDM, and NAFLD was retrieved from the raw data. A new variable, AIP, was calculated for this study, AIP = log10 (TG/HDL).

#### 2.3.1 Exposure variables and outcome variable

The exposure variable was estimated using AIP, calculated as log10 (TG/HDL) (28). We analyzed AIP as a continuous variable to explore the relationship between each 0.1 unit change in AIP (AIP*10) and NAFLD. Based on AIP tertiles, participants were divided into three groups: T1 (<0.15, *n* = 195), T2 (0.15–0.32, *n* = 195), and T3 (>0.32, *n* = 196). The outcome variable was NAFLD, diagnosed by ultrasonography. Diagnosis of fatty liver was based on the presence of at least two out of three abnormal findings: diffuse echogenic enhancement (“bright”), liver echo that was stronger than the renal parenchyma, as well as ambiguity and narrowing of the vascularized hepatic vein lumen (29). Furthermore, other liver diseases were excluded from consideration.

#### 2.3.2 Covariates

A thorough method was employed to identify the risk factors linked to NAFLD from clinical expertise, original studies, and existing literature. Given the preceding factors, the covariates utilized included age, AST, previous experience of childbirth, ALT, pre-pregnancy BMI, GGT, TC, GDM, LDL-C, FPG, and homeostasis model assessment-insulin resistance (HOMA-IR). The formula for calculating HOMA-IR is given: [FPG (mmol/L) × insulin (μU/ml)/22.5]. This formula is widely used in research and clinical settings to estimate insulin resistance based on fasting plasma glucose (FPG) and fasting insulin levels. It is a straightforward method that helps in assessing the function of insulin in maintaining glucose homeostasis.

### 2.4 Statistical analysis

This study was completed by our team in the First People’s Hospital of Changde City over a period of 5 months. To analyze the distribution of baseline data, participants were divided into two groups based on their NAFLD status. Continuous variables were presented as mean and standard deviation (SD) for those with a normal distribution and as median and interquartile range (25th–75th percentiles) for skewed distributions. Categorical variables were expressed as frequency and percentage. To compare variability between groups, we used one-way analyses of variance for variables with a normal distribution, the Kruskal-Wallis test for variables with skewed distributions, and the chi-square test for categorical variables.

In this study, logistic regression analysis (which is a statistical model that predicts the probability of an event occurring in a dichotomous problem) was used to build four different models to determine the odds ratio (OR) and 95% confidence interval (95% CI) for AIP and NAFLD. Model 1 excluded all confounding factors, while Model 2 corrected for socio-demographic characteristics such as nulliparity, age, and pre-pregnancy BMI. Model 3 was further adjusted for AST, ALT, GGT, TC, and LDL, and Model 4 represented a fully adjusted version, incorporating FPG, HOMA-IR, and GDM. The study assessed the risk of NAFLD using adjusted OR and 95% CI. AIP was stratified into tertiles, and the *p* value for trends was calculated. In addition, restricted cubic spline (RCS) regressions were performed on the 5th, 35th, 65th, and 95th percentiles of AIP after adjusting for the variables in model 4 to assess linearity and test the dose–response curve between AIP and NAFLD.

To evaluate the robustness of our findings, we conducted several sensitivity analyses. Participants with pre-pregnancy BMI ≥25 kg/m^2^ (30) or GDM were excluded from sensitivity testing (31). Furthermore, we conducted subgroup analyses (dividing study participants into subgroups based on specific characteristics, assessing the effects of each subgroup separately, and comparing the differences in effects between these subgroups), stratifying by age, nulliparity, pre-pregnancy BMI, HOMA-IR, and GDM, to examine the relationship between AIP and NAFLD across different subgroups. These sensitivity and subgroup analyses were aimed at ensuring the stability of our results.

The Free Statistics analytic platform (version 2.0, Beijing, China, http://www.clinicalscientists.cn/freestatistics) and the R statistical program (version 4.2.2, http://www.R-project.org, The R Foundation) were used for all analyses. A two-sided *p* value<0.05 was considered statistically significant.

## 3 Results

### 3.1 Characteristics of participants

586 pregnant women who met the study’s eligibility requirements had an average age of 32.07 ± 3.77, with the youngest 22 years and the oldest 43 years. NAFLD was present in 110 instances (18.8%) of the pregnant women, and the AIP was evaluated at 0.22 (0.11, 0.37). With the data split into two groups according to whether or not NAFLD was present, the baseline characteristics of the pregnant women are shown in Table 1. The results showed that the AIP of NAFLD patients (0.33 [0.18, 0.46]) was higher than that of non-NAFLD patients (0.21 [0.10, 0.35]). The incidence of GDM was found to be higher in individuals with NAFLD.

**Table 1:** Baseline characteristics of participants.

Characteristics	Total (*n* = 586)	Without NAFLD (*n* = 476)	With NAFLD (*n* = 110)	*p*-value
Age (year)	32.07 ± 3.77	32.18 ± 3.65	31.58 ± 4.26	0.131
Parity				0.937
No	307 (52.39)	249 (52.31)	58 (52.73)	
Yes	279 (47.61)	227 (47.69)	52 (47.27)	
BMI (kg/m^2^)				<0.001
<25	491 (83.79)	422 (88.66)	69 (62.73)	
≥25	95 (16.21)	54 (11.34)	41 (37.27)	
AST(IU/L)				0.126
<40	579 (98.81)	472 (99.16)	107 (97.27)	
≥40	7 (1.19)	4 (0.84)	3 (2.73)	
ALT(IU/L)				0.031
<40	572 (97.61)	468 (98.32)	104 (94.55)	
≥40	14 (2.39)	8 (1.68)	6 (5.45)	
GGT(IU/L)				<0.001
<22.5	535 (91.30)	446 (93.7)	89 (80.91)	
≥22.5	51 (8.70)	30 (6.3)	21 (19.09)	
TC (mg/dl)				0.661
<200	498 (84.98)	406 (85.29)	92 (83.64)	
≥200	88 (15.02)	70 (14.71)	18 (16.36)	
TG (mg/dl)				<0.001
<150	461 (78.67)	390 (81.93)	71 (64.55)	
≥150	125 (21.33)	86 (18.07)	39 (35.45)	
HDL (mg/dl)				<0.001
<55	142 (24.23)	102 (21.43)	40 (36.36)	
≥55	444 (75.77)	374 (78.57)	70 (63.64)	
LDL (mg/dl)				0.441
<130	575 (98.12)	468 (98.32)	107 (97.27)	
≥130	11 (1.88)	8 (1.68)	3 (2.73)	
GDM				<0.001
No	550 (93.86)	460 (96.64)	90 (81.82)	
Yes	36 (6.14)	16 (3.36)	20 (18.18)	
HOMA-IR	1.89 ± 1.79	1.72 ± 1.73	2.61 ± 1.88	<0.001
AIP	0.22 (0.11, 0.37)	0.21 (0.10, 0.35)	0.33 (0.18, 0.46)	<0.001

NAFLD, nonalcoholic fatty liver disease; parity (No: Never given birth; Yes: Have given birth); BMI: body mass index; AST, aspartate aminotransferase; ALT, alanine aminotransferase; GGT, gamma-glutamyl transferase; TC, total cholesterol; TG, triglyceride; HDL, high-density lipoprotein cholesterol; LDL, low-density lipid cholesterol; FPG, fasting plasma glucose; HOMA-IR, homeostasis model assessment-insulin resistance; GDM, gestational diabetes mellitus; AIP, atherogenic index of plasma.

### 3.2 Association between AIP and NAFLD

**Figure 2:**
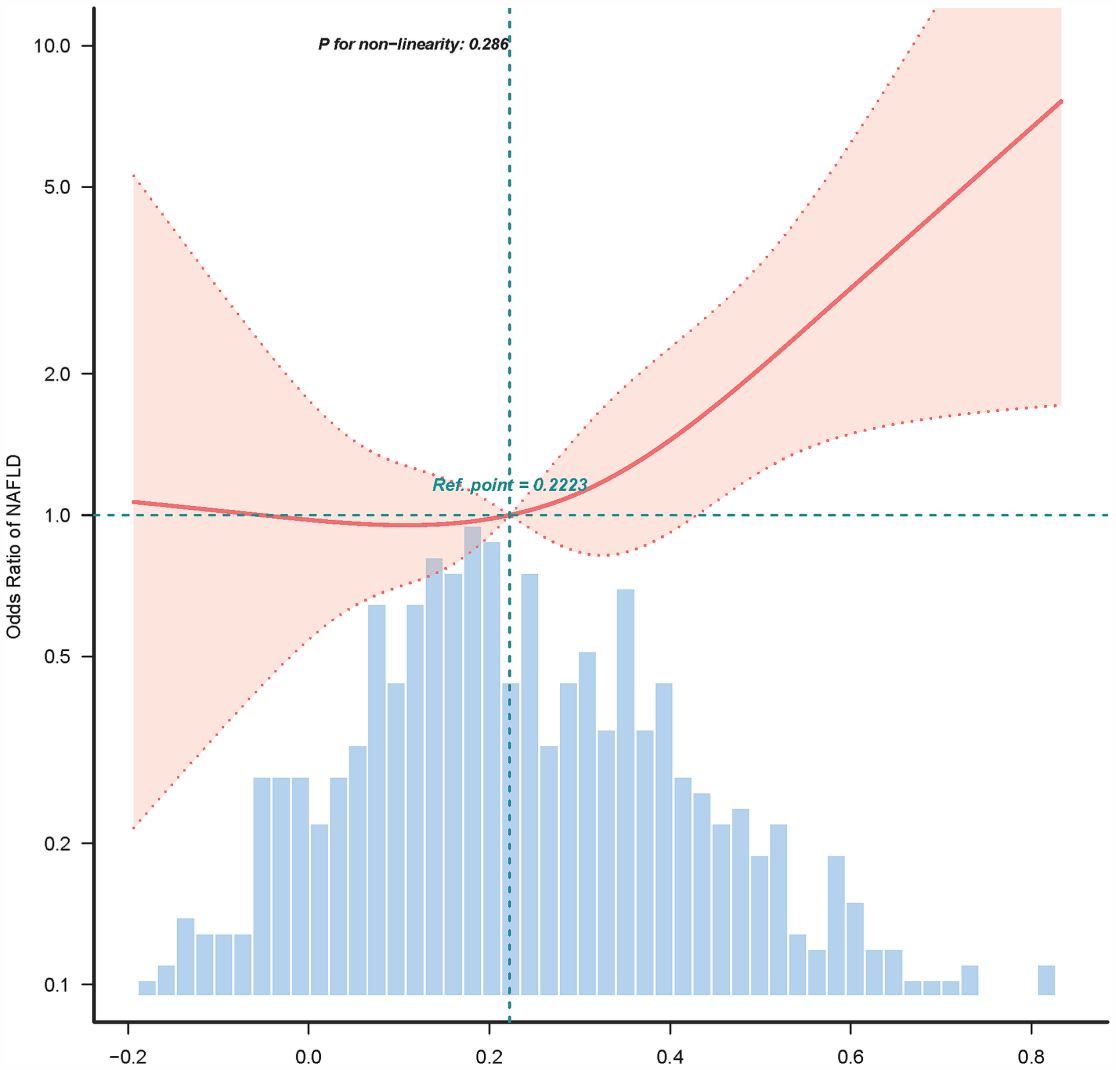
Association between AIP and NAFLD in RCS.

The pattern of linearity between AIP and NAFLD is evident in Figure 2.

Supplementary Table S1 presents the outcomes of univariate analysis, revealing a positive correlation between NAFLD risk and pre-pregnancy BMI, ALT, GGT, TG, FPG, insulin, HOMA-IR, GDM, and AIP. Conversely, HDL showed an inverse association with NAFLD risk.

In Table 2, the multivariable logistic regression model reveals a positive association between AIP and NAFLD. In Model 1, AIP exhibited a positive association with NAFLD, with an OR of 1.33 (95% CI: 1.19–1.48, *p* < 0.001) without adjusting for any covariates. In Model 2, after adjusting for age, nulliparity, and pre-pregnancy BMI, consistent results were obtained with no significant differences (OR: 1.25, 95% CI: 1.11–1.4, *p* < 0.001). Furthermore, in Model 3, after adjusting for AST, ALT, GGT, TC, and LDL, the results still demonstrated a positive association (OR: 1.24, 95% CI: 1.11–1.4, *p* < 0.001). Model 4, which further adjusted for FPG, HOMA-IR, and GDM, continued to show a positive association between AIP and NAFLD (OR: 1.2, 95% CI: 1.06–1.37, *p* < 0.001). This suggested that after accounting for potential confounders in Model 4, each 0.1 unit increase in AIP was associated with a 20% increase in NAFLD risk. Correlations remained when AIP as a categorical variable was divided into tertiles, T1 (<0.16, *n* = 195), T2 (0.16–0.32, *n* = 195), and T3 (>0.32, *n* = 196). Individuals in T3 (AIP > 0.32) had an OR of 2.02 (95% CI: 1.11–3.68, *p* = 0.021) compared to individuals in the lowest tertile of AIP, T1 (AIP < 0.15). This result suggests a 2.02-fold increased risk of NAFLD in pregnancy in the T3 group compared to the T1 group.

**Table 2:** Relationship between AIP and NAFLD in in Korean pregnant women.

Variable	Model 1 OR (95%CI) *p* value		Model 2 OR (95%CI) *p* value		Model 3 OR (95%CI) *p* value		Model 4 OR (95%CI) *p* value	
AIP*10	1.33 (1.19 ~ 1.48)	<0.001	1.25 (1.11 ~ 1.4)	<0.001	1.24 (1.11 ~ 1.4)	<0.001	1.2 (1.06 ~ 1.37)	0.004
AIP								
T1(<0.15)	1(Ref)		1(Ref)		1(Ref)		1(Ref)	
T2(0.15–0.32)	1.15 (0.63 ~ 2.06)	0.652	0.97 (0.51 ~ 1.83)	0.914	0.91 (0.48 ~ 1.75)	0.787	0.9 (0.47 ~ 1.72)	0.744
T3(>0.32)	3.07 (1.82 ~ 5.19)	<0.001	2.41 (1.36 ~ 4.27)	0.003	2.26 (1.26 ~ 4.05)	0.006	2.02 (1.11 ~ 3.68)	0.021
*p* for Trend		<0.001		<0.001		0.002		0.012

Model 1 was adjusted for nothing. Model 2 was adjusted for the variables age, pre-pregnancy BMI and Nulliparity. Model 3 was adjusted for all covariates in model 2+ AST, ALT, GGT, TC and LDL. Model 4 was adjusted for all covariates in model 3+ FPG, HOMA-IR, and GDM. AIP*10: AIP (per 0.1 unit). OR, odds ratios; CI, confidence interval; Ref, Reference.

### 3.3 Sensitivity analysis

To test our conclusions’ robustness, we also performed several sensitivity analyses. According to Table 3, the initial sensitivity examination focused on individuals with a pre-pregnancy BMI of less than 25 kg/m^2^. Despite considering additional factors, we found a significant association correlation between AIP and NAFLD (OR: 1.24, 95% CI: 1.08–1.44). A similar sensitivity analysis on participants without GDM also demonstrated a positive relationship between AIP and the risk of NAFLD (OR: 1.21, 95% CI: 1.05–1.39), after adjusting for other variables (Table 3). These sensitivity analyses confirmed the reliability of our findings.

**Table 3:** Relationship between AIP and NAFLD in different sensitivity analyses.

Variable	Model I (OR, 95% CI, *p*)	Model II (OR, 95% CI, *p*)
AIP*10	1.24 (1.08 ~ 1.44)0.003	1.21 (1.05 ~ 1.39)0.007
AIP		
T1(<0.15)	1(Ref)	1(Ref)
T2(0.15–0.32)	1.02 (0.5 ~ 2.08)0.962	0.82 (0.42 ~ 1.6)0.57
T3(>0.32)	2.18 (1.13 ~ 4.2)0.02	2.05 (1.12 ~ 3.76)0.021
*p* for Trend	0.017	0.012

Model I was sensitivity analysis after excluding those with pre-pregnancy BMI ≥ 25 kg/m^2^, *n* = 490. We adjusted for Age, Nulliparity, AST, ALT, GGT, TC, LDL, FPG, HOMA-IR, and GDM. Model II was a sensitivity analysis after excluding those with GDM, *n* = 550. We adjusted for Age, pre-pregnancy BMI, Nulliparity, AST, ALT, GGT, TC, LDL, FPG and HOMA-IR. AIP*10: AIP (per 0.1 unit).

### 3.4 Subgroup analysis

**Figure 3:**
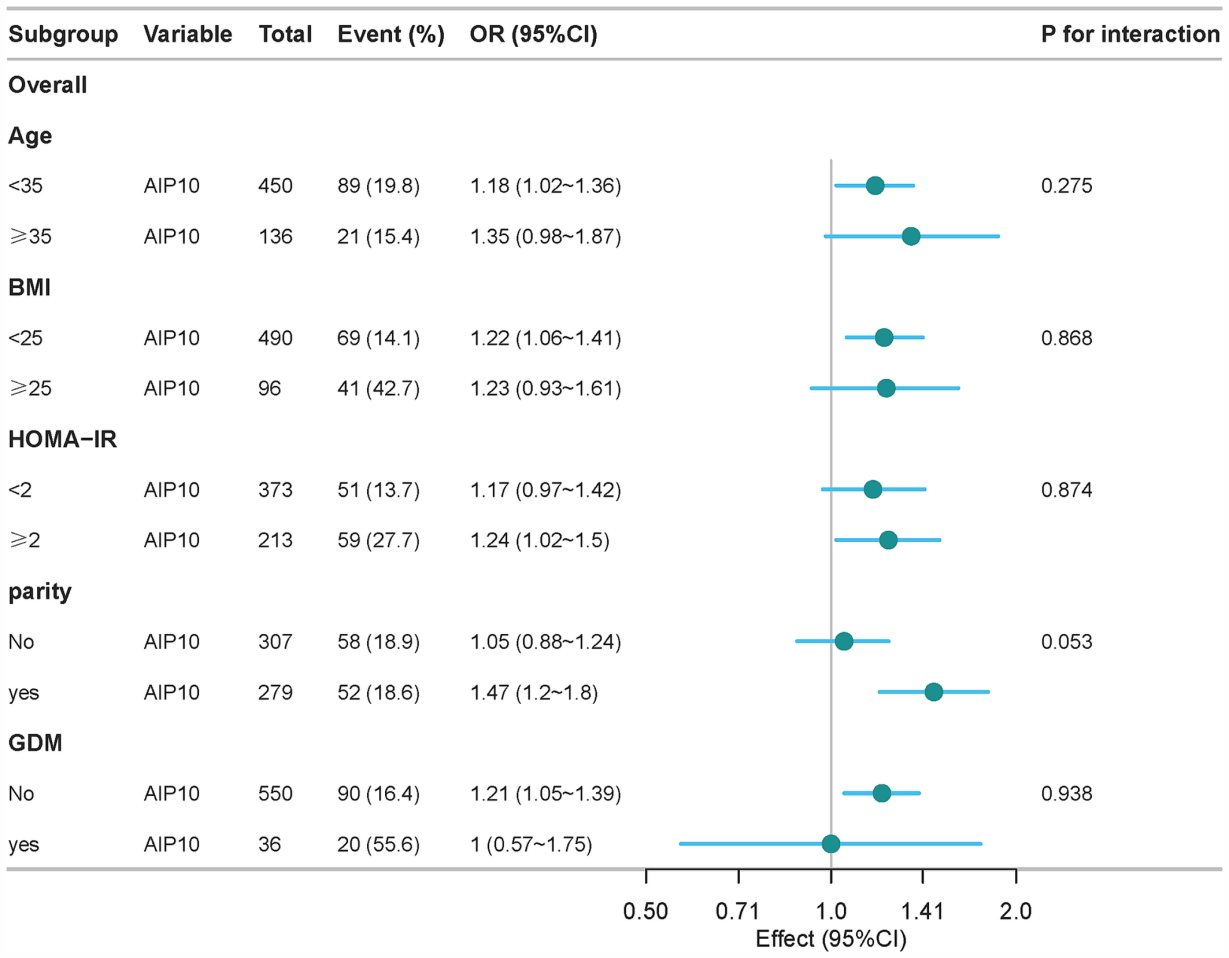
Subgroup analysis of the association between AIP and NAFLD was performed based on baseline characteristics.

The association between AIP and NAFLD was consistent across various subgroups, remaining robust irrespective of factors such as age, pre-pregnancy BMI, HOMA-IR, nulliparity, and GDM (Figure 3). This consistency underscores the strength and reliability of our results, suggesting that the relationship between AIP and NAFLD is not influenced by these potential confounding variables.

## 4 Discussion

Univariate regression analysis showed that NAFLD was strongly correlated with pre-pregnancy BMI, HOMA-IR, GDM and AIP. Among these, AIP was found to be an indicator of insulin resistance, which has a strong correlation with diabetes, cardiovascular and metabolic diseases (32–34), and it is reliable and easy to obtain. Previous studies have shown that AIP is associated with NAFLD, but no study has included pregnant women. Therefore, we investigated the association between AIP and NAFLD in the Korean pregnant population. In this secondary analysis of a prospective cohort study, AIP was associated with NAFLD after adjusting for confounding factors. It found that for every 0.1 unit increase in the AIP, there was a 20% rise with the risk of NAFLD. The positive association between AIP and NAFLD was further strengthened by the sensitivity and subgroup analysis results, demonstrating the results’ stability.

With the escalation of the global standard of living, NAFLD has emerged as the most prevalent hepatic disorder worldwide (5). A recent epidemiological study conducted in Asia has revealed a persistent upward trend in the prevalence of NAFLD among Korean women. The study, which was conducted in 2019, documented a 22.42% prevalence rate among the female population (35), inclusive of women of childbearing age. This phenomenon poses a significant threat to the reproductive safety of women, necessitating further investigation and vigilant monitoring. The main features of this disease are abnormal lipid metabolism and insulin resistance. Fat metabolism in pregnant women is a complex and dynamic process that can be divided into two main phases: the anabolic phase in early pregnancy and the catabolic phase in late pregnancy. In early pregnancy, maternal insulin sensitivity increases, stimulating fat synthesis and leading to the accumulation of subcutaneous fat as an energy reserve for fetal development (36). In mid-pregnancy, triglyceride levels rise significantly, with the liver synthesising triglycerides from circulating free fatty acids (37). In late pregnancy, however, maternal insulin resistance increases, inhibiting fat synthesis and promoting lipolysis. Hormones released from the placenta, such as human placental lactogen and human placental growth hormone, further regulate fat metabolism, resulting in increased release of free fatty acids into the bloodstream for energy production or triglyceride re-synthesis (38, 39). Overall, changes in lipid metabolism during pregnancy not only support fetal growth, but also affect maternal health and pregnancy outcomes.

Previous studies have indicated that pregnant women with NAFLD are at higher risk of adverse pregnancy outcomes, including GDM, pre-eclampsia, miscarriage, and preterm labour (31, 40).NAFLD is usually detected by ultrasound, which is not part of a routine pregnancy test. Physicians may be more concerned about pregnant women who have a high pre-pregnancy BMI or liver function abnormalities (25, 41, 42), which can lead some patients to miss the diagnosis and experience adverse pregnancy outcomes.

According to our study, AIP is independently associated with NAFLD in pregnant women, consistent with the results of previous studies conducted in the general population. Liu et al. found a significant association between AIP and NAFLD in a Beijing physical examination population (23). Dong et al. reported a 50.84-fold increase in the risk of NAFLD for each 1-SD increase in AIP in a non-obese population (43). Xie et al. found that AIP had a higher risk of fatty liver compared to other parameters, such as BMI, ALT, and AST, with an OR of 13.992 (44). The difference with their study is that our study population was pregnant women, and due to the series of changes that occur in the mother’s body during pregnancy in order to nurture the fetus, our results showed that AIP was still positively associated with NAFLD. We also did further sensitivity analyses to support the existence of this association in a population of pregnant Korean women with a body mass index less than 25 kg/m_2_ or without gestational diabetes mellitus. Subgroup analysis confirmed that the relationship between AIP and NAFLD was unchanged in different strata. A study on the prediction model of NAFLD during pregnancy in Sri Lanka showed that fatty liver index was a valid predictor and could be used as a screening tool for NAFLD (45). Our study found an association between AIP and NAFLD, which is expected to be added to the prediction model for further analysis.

The AIP has emerged as a more robust predictor of metabolic disorders compared to traditional measures such as BMI and HOMA-IR. Research indicates that AIP demonstrates superior predictive value, particularly among women and younger populations, where BMI fails to account for fat distribution and its implications for insulin resistance (46). Studies have shown that AIP correlates more strongly with lipid profiles and metabolic markers, making it a useful indicator of coronary artery disease risk. In younger demographics, the prevalence of insulin resistance is often underestimated by BMI alone, underscoring AIP’s sensitivity as a marker in this group (47).

NAFLD is a leading cause of cirrhosis and can potentially progress to hepatocellular carcinoma (48). The pathogenesis of NAFLD is complex, involving factors such as insulin resistance, accumulation of fatty acids, production of free radicals, oxidative stress, and inflammation (49). NAFLD is not just a liver disease, as multiple organs and tissues play a role in its development (50). Impaired secretion of adipokines by adipose tissue, which promotes lipogenesis, is a critical factor. These processes collectively lead to the development of hepatic inflammation and fibrosis (51, 52). AIP, which is the logarithm of the ratio of triglycerides to HDL cholesterol, reflects the balance between triglycerides and HDL and is a more sensitive screening test for NAFLD than lipid markers alone.

Our study has the following strengths. Most notably, we demonstrated for the first time a correlation between AIP levels and NAFLD in pregnancy. Second, we considered confounding factors as much as possible. Third, we performed sensitivity analyses to assess the reliability of our conclusions, such as transforming AIP into a categorical variable, reassessing the relationship between AIP and NAFLD by excluding individuals with BMI ≥25 kg/m^2^ or GDM, performing subgroup examinations, and finding that the results were stable across subgroups.

The research has several limitations. Firstly, as our study was a secondary analysis, data on AIP and NAFLD were obtained simultaneously in the original study. In the future, we can do further studies to exclude participants with pre-existing NAFLD prior to gestation. Secondly, NAFLD diagnosis was based on ultrasonography instead of the gold standard liver biopsy (53), which is not suitable for pregnant women. The diagnostic efficacy of ultrasound decreases when the liver has <15% steatosis (54). Thirdly we excluded participants with no previous history of chronic liver disease (such as hepatitis B or C, autoimmune hepatitis, or primary biliary cholangitis) by collecting clinical information rather than by blood tests. Fourth, we did not take into account the effect of gestational age on NAFLD. Fifth, only one investigator retrieved the secondary data, but later all members of our team worked together to re-download the data and check the original data. Lastly, our study was not validated in other ethnic groups of pregnant women, so in future studies, we will further expand the population for analysis.

## 5 Conclusion

The results of this secondary analysis of a prospective cohort study indicate a positive association between AIP and NAFLD in a group of Korean pregnant women. These findings may aid in creating screening methods for NAFLD in pregnant women.

## Data Availability

Publicly available datasets were analyzed in this study. This data can be found here: the “Fatty Liver in Pregnancy” registry (clinicaltrials.gov registration no. NCT02276144), establishedby Korean researchers. https://journals.plos.org/plosone/article?id=10.1371/journal.pone.0221400.
