# Supplementary figures and images for: Association between the atherogenic index of plasma and non-alcoholic fatty liver disease in Korean pregnant women: secondary analysis of a prospective cohort study
Source: Front Nutr. 2025 Jan 31;12:1511952. doi: 10.3389/fnut.2025.1511952 (PMC11825326; doi:10.3389/fnut.2025.1511952)

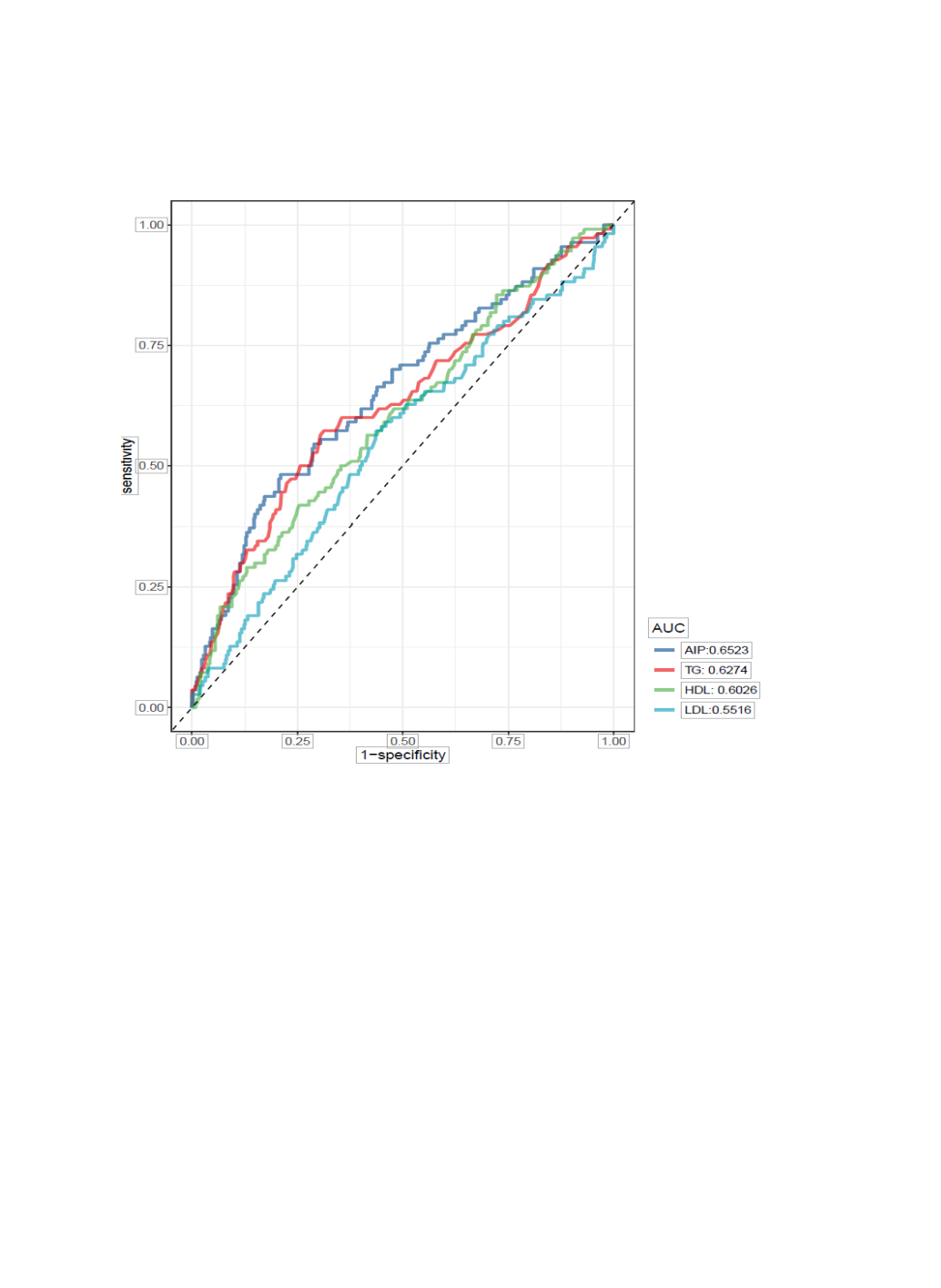

Supplement: Supplementary file 1 [file Image_1.TIF]
