# Supplementary material for: Association between the atherogenic index of plasma and non-alcoholic fatty liver disease in Korean pregnant women: secondary analysis of a prospective cohort study
Source: Front Nutr. 2025 Jan 31;12:1511952. doi: 10.3389/fnut.2025.1511952 (PMC11825326; doi:10.3389/fnut.2025.1511952)
Supplement: Supplementary file 2 [file Table_1.DOCX]

TableS1 The results of univariate analysis

| Variable | OR_95CI | *P*_value |
| --- | --- | --- |
| Age(year) | 0.96 (0.91~1.01) | 0.131 |
| parity | 0.98 (0.65~1.49) | 0.937 |
| Pre-pregnancy BMI(kg/m^2^) | 1.3 (1.22~1.39) | <0.001 |
| AST (IU/L) | 1.02 (1~1.04) | 0.11 |
| ALT (IU/L) | 1.04 (1.02~1.06) | <0.001 |
| GGT (IU/L) | 1.03 (1.01~1.05) | 0.008 |
| TC(mg/dL) | 1.01 (1~1.01) | 0.174 |
| TG(mg/dL) | 1.01 (1.01~1.01) | <0.001 |
| HDL(mg/dL) | 0.97 (0.96~0.99) | 0.001 |
| LDL(mg/dL) | 1.01 (1~1.02) | 0.108 |
| FPG(mg/dL) | 1.03 (1.01~1.05) | 0.01 |
| insulin(μIU/mL) | 1.09 (1.06~1.13) | <0.001 |
| HOMA-IR | 1.38 (1.19~1.6) | <0.001 |
| GDM | 6.39 (3.19~12.8) | <0.001 |
| AIP*10 | 1.33 (1.19~1.48) | <0.001 |

Abbreviations: OR, odds ratio; CI, confidence interval
